# Supplementary material for: The Small RNA Universe of Capitella teleta
Source: Front Mol Biosci. 2022 Feb 25;9:802814. doi: 10.3389/fmolb.2022.802814 (PMC8915122; doi:10.3389/fmolb.2022.802814)
Supplement: Supplementary file 1 [file DataSheet1.ZIP › Supplement/known/CAPTEscaffold_14_1879.pdf]

Provisional ID	:	CAPTEscaffold_14_1879
Score total	:	147546.6
Score for star read(s)	:	3.9
Score for read counts	:	147539.4
Score for mfe	:	2.3
Score for randfold	:	1.6
Score for cons. seed	:	-0.6
Total read count	:	289404
Mature read count	:	203050
Loop read count	:	0
Star read count	:	86354

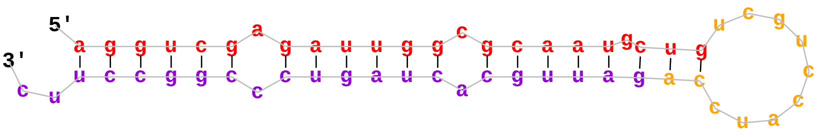

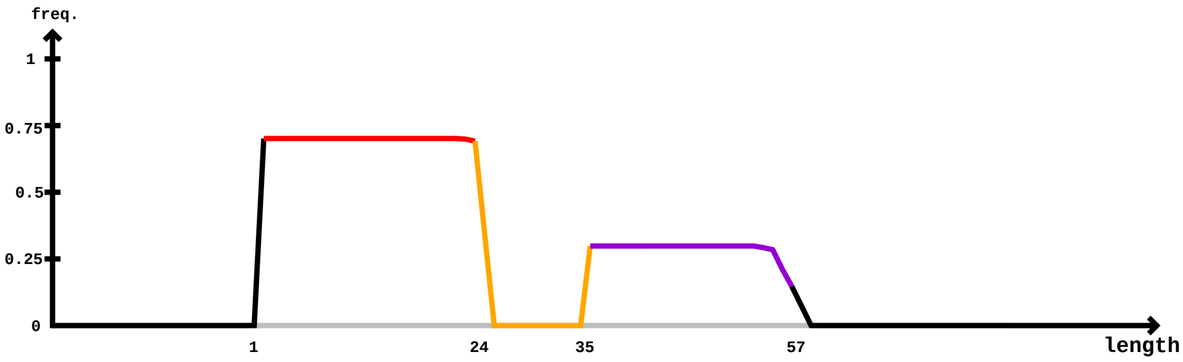

Mature **Star**

```
5'- gcguguugcucggugccagaaggucgagauuggcgcaaugcugucguccauccagauugcacuagucccggccuucuggcucugcugcucgucuucaucucgccucggcgacu  -3'   obs
    gcguguugcucggugccagaaggucgagauuggcgcaaugcugucguccauccagauugcacuagucccggccuucuggcucugcugcucgucuucaucucgccucggcgacu        exp
    gcguguugcucggugccagaaggucgagauuggcgcaaugcugucguccauccagauugcacuagucccggccuucuggcucugcugcucgucuucaucucgccucggcgacu        known
    (((.((.((..((.(((((((((((.((((((.(((((.(((.........)))))))).)))))).))))))))))).)))).)).)))........((((....))))..     reads    mm      sample

    gcguguugcucggugccaga.............................................................................................     1        0       seq
    .cguguugcucggugccaga.............................................................................................     9        0       seq
    ..guguugcucggugccaga.............................................................................................     2        0       seq
    ...............ccagaaggucgagauuggcgc.............................................................................     1        0       seq
    .................agaaggucgagauuggcg..............................................................................     1        0       seq
    ...................aaggucgagauuggcgcaaugc........................................................................     2        0       seq
    ...................aaggucgagauuggcgcaaugcu.......................................................................     32       0       seq
    ...................aaggucgagauuggcgcaaugcug......................................................................     17       0       seq
    ...................aaggucgagauuggcgcaaugcuU......................................................................     1        1       seq
    ...................Caggucgagauuggcgcaaugcug......................................................................     1        1       seq
    ....................aggucgagauuggcgcaa...........................................................................     3        0       seq
    ....................aggucgagauuggcgcaau..........................................................................     1        0       seq
    ....................aggucgagauuggcgcaaug.........................................................................     17       0       seq
    ....................Uggucgagauuggcgcaaugc........................................................................     1        1       seq
    ....................aggucgagaAuggcgcaaugc........................................................................     1        1       seq
    ....................aggucgagauuggcgcaaugc........................................................................     663      0       seq
    ....................aggucgagauuggcgcaaugA........................................................................     1        1       seq
    ....................aggucgagauuUgcgcaaugc........................................................................     1        1       seq
    ....................aggucgagauuggcgAaaugc........................................................................     1        1       seq
    ....................aggucgGgauuggcgcaaugc........................................................................     1        1       seq
    ....................Gggucgagauuggcgcaaugc........................................................................     10       1       seq
    ....................aggucgagauuggAgcaaugc........................................................................     1        1       seq
    ....................aggucgagauuAgcgcaaugc........................................................................     1        1       seq
    ....................aggucAagauuggcgcaaugc........................................................................     2        1       seq
    ....................Gggucgagauuggcgcaaugcu.......................................................................     42       1       seq
    ....................Cggucgagauuggcgcaaugcu.......................................................................     1        1       seq
    ....................aggucgagaCuggcgcaaugcu.......................................................................     1        1       seq
    ....................agguAgagauuggcgcaaugcu.......................................................................     1        1       seq
    ....................aggucgagauuggcgcaaugcA.......................................................................     4        1       seq
    ....................aggucAagauuggcgcaaugcu.......................................................................     1        1       seq
    ....................aggucgagauuggcgcaaugcG.......................................................................     8        1       seq
```

Mature **Star**

gcguguugcucggugccagaaggucgagauuggcgcaaugcugucguccauccagauugcacuagucccggccuucuggcucugcugcucgucuucaucucgccucggcgacu			
....................aggucgagauuggUgcaaugcu...	1	1	seq
....................aggucgagauuAgcgcaaugcu...	1	1	seq
....................aggucgagauuggcgcaaugcu...	2070	0	seq
....................aggucgagauuCgcgcaaugcu...	1	1	seq
....................aggucgaAauuggcgcaaugcu...	1	1	seq
....................aggucgagauugAcgcaaugcu...	2	1	seq
....................agNucgagauuggcgcaaugcu...	1	1	seq
....................aggucgagGuuggcgcaaugcu...	1	1	seq
....................aggucgagauuggcgGaaugcu...	1	1	seq
....................aggucgagauuggcgcaaugAu...	1	1	seq
....................Nggucgagauuggcgcaaugcu...	1	1	seq
....................aggucgagauAggcgcaaugcug..	80	1	seq
....................aggucCagauuggcgcaaugcug..	8	1	seq
....................aggucgNgauuggcgcaaugcug..	1	1	seq
....................aggucgagaAuggcgcaaugcug..	43	1	seq
....................aggucgagauuggcgcaaugcuN..	2	1	seq
....................aggucgagauuggGgcaaugcug..	16	1	seq
....................aggGcgagauuggcgcaaugcug..	8	1	seq
....................aggucgagauuCgcgcaaugcug..	8	1	seq
....................aggucgagGuuggcgcaaugcug..	30	1	seq
....................aggCcgagauuggcgcaaugcug..	16	1	seq
....................agUucgagauuggcgcaaugcug..	2	1	seq
....................aggucgagauuggcgcaaugcug..	196792	0	seq
....................aggucgagauuggcgcaaCgcug..	24	1	seq
....................aggucgagauuggcgcaaGgcug..	13	1	seq
....................aggucgagauuggcgcaaugGug..	5	1	seq
....................aggucgagauuggcgcaauUcug..	10	1	seq
....................agAucgagauuggcgcaaugcug..	29	1	seq
....................agguUgagauuggcgcaaugcug..	92	1	seq
....................aggucgaAauuggcgcaaugcug..	15	1	seq
....................aAgucgagauuggcgcaaugcug..	104	1	seq
....................Nggucgagauuggcgcaaugcug..	59	1	seq
....................aggucgagauuggcgcaUugcug..	13	1	seq
....................aggucgagauuggcgcaaugcuC..	15	1	seq
....................aggucgagauuggcgGaaugcug..	11	1	seq
....................aggucgagauuUgcgcaaugcug..	14	1	seq
....................agCucgagauuggcgcaaugcug..	20	1	seq
....................aggucgagauuggcgcaGugcug..	29	1	seq
....................aggucgagaGuggcgcaaugcug..	4	1	seq
....................aggucgaCauuggcgcaaugcug..	6	1	seq
....................Gggucgagauuggcgcaaugcug..	865	1	seq
....................aggucgagauuggUgcaaugcug..	51	1	seq
....................aggucgagauugUcgcaaugcug..	8	1	seq
....................aggucgUgauuggcgcaaugcug..	24	1	seq
....................aggucgaUauuggcgcaaugcug..	12	1	seq
....................aggucgagauuggcgcaauAcug..	22	1	seq
....................aggucgagauCggcgcaaugcug..	38	1	seq
....................aggucgagauuggcgcaauCcug..	7	1	seq
....................aggucgagauuggcUcaaugcug..	15	1	seq
....................aggucgagauGggcgcaaugcug..	6	1	seq
....................agguGgagauuggcgcaaugcug..	11	1	seq
....................aggucgagauuAgcgcaaugcug..	135	1	seq
....................aggucgagauuggcgcaaugcCg..	19	1	seq
....................aggucgagauuggcgcaaugcuA..	35	1	seq
....................aggucgagauuggcgcaauNcug..	1	1	seq
....................aggucUagauuggcgcaaugcug..	13	1	seq
....................aggucgCgauuggcgcaaugcug..	4	1	seq
....................aggucgagauugAcgcaaugcug..	23	1	seq
....................aggucgagauuggcAcaaugcug..	43	1	seq
....................aggucgagauugCcgcaaugcug..	10	1	seq
....................Uggucgagauuggcgcaaugcug..	16	1	seq
....................aggucgagauuggcgcUaugcug..	28	1	seq
....................aggucgagauuggcgcaaugAug..	50	1	seq
....................aggucgagauuggcgcNaugcug..	1	1	seq
....................aNgucgagauuggcgcaaugcug..	7	1	seq
....................aggucgagauuggAgcaaugcug..	63	1	seq
....................aggAcgagauuggcgcaaugcug..	74	1	seq
....................aggucgagauuggcgUaaugcug..	17	1	seq
....................aggucgagauuggcgAaaugcug..	23	1	seq
....................aggucgagauuggcgcaaugUug..	11	1	seq

Mature / Star			
gcguguugcucggugccagaaggucgagauuggcgcaaugcugucguccauccagauugcacuagucccggccuucuggcucugcugcucgucuucaucucgccucggcgacu			
....................aggucgagauuggcgNaaugcug..	1	1	seq
....................agNucgagauuggcgcaaugcug..	1	1	seq
....................aggucgagUuuggcgcaaugcug..	16	1	seq
....................aggucgagCuuggcgcaaugcug..	1	1	seq
....................aggucgagauuggcgcGaugcug..	33	1	seq
....................aggucgagauuggcgcaaugcAg..	35	1	seq
....................aggucAagauuggcgcaaugcug..	221	1	seq
....................Cggucgagauuggcgcaaugcug..	9	1	seq
....................aggucgagauuggcgcaaugcuU..	17	1	seq
....................aggucgagauuggcCcaaugcug..	6	1	seq
....................aggucNagauuggcgcaaugcug..	4	1	seq
....................aggucgagaCuggcgcaaugcug..	17	1	seq
....................aUgucgagauuggcgcaaugcug..	14	1	seq
....................aggucgagauuggcgcaaAgcug..	34	1	seq
....................agguAgagauuggcgcaaugcug..	38	1	seq
....................aggucgagauuggcgcaaugcGg..	437	1	seq
....................aCgucgagauuggcgcaaugcug..	13	1	seq
....................aggucgGgauuggcgcaaugcug..	34	1	seq
....................aggucgagauuggcgcaaugcugu...	37	0	seq
....................aggucgagauuggcgcaaugcuUu...	1	1	seq
....................aggucgagauuggcgcaaugcugG...	5	1	seq
....................agAucgagauuggcgcaaugcugu...	1	1	seq
....................aggucgagauuggcgcaaugcugC...	1	1	seq
....................aggucgagauuggcgcaaugcugA...	22	1	seq
....................Gggucgagauuggcgcaaugcugu...	1	1	seq
....................aggucgagauuggcgcaaugcuguU..	1	1	seq
....................aggucgagauuggcgcaaugcugucguc...	1	0	seq
....................aggucgagauuggcgcaaugcugucgucU..	1	1	seq
.....................ggGcgagauuggcgcaaugcug..	1	1	seq
.....................ggucgagauuggcgcaaugcGg..	1	1	seq
.....................ggucgagauuggcgcaaugcug..	21	0	seq
.....................Agucgagauuggcgcaaugcug..	1	1	seq
......................gucgagauuggcgcaaugcug..	21	0	seq
.......................ucgagauuggcgcaaugcug..	2	0	seq
........................cgagauuggcgcaaugcug..	4	0	seq
.........................gagauuggcgcaaugcug..	1	0	seq
....Cgauugcacuagucccggccuu......................................	1	1	seq
....gauGgcacuagucccggc...	1	1	seq
....gGuugcacuagucccggc...	8	1	seq
....gauugcacuaguccGggc...	1	1	seq
....gauugcacuagucccggc...	1603	0	seq
....gaAugcacuagucccggc...	2	1	seq
....Uauugcacuagucccggc...	1	1	seq
....gauugcacuagucccggA...	101	1	seq
....gauugcacuagCcccggc...	2	1	seq
....gauugcacuUguccggc...	2	1	seq
....gauugcacuagucccAgc...	1	1	seq
....gauugcacuagucAcggc...	2	1	seq
....gauugAacuagucccggc...	2	1	seq
....Aauugcacuagucccggc...	4	1	seq
....Uauugcacuagucccggcc..	1	1	seq
....gauugAacuagucccggcc..	1	1	seq
....gauugcGcuagucccggcc..	3	1	seq
....gauugcUcuagucccggcc..	1	1	seq
....gaCugcacuagucccggcc..	2	1	seq
....gauAgcacuagucccggcc..	1	1	seq
....gauugcacuaAucccggcc..	1	1	seq
....gauugcacuagucccAgcc..	1	1	seq
....gauugcacuagucccgAcc..	1	1	seq
....gauugcacuagucccUgcc..	1	1	seq
....gGuugcacuagucccggcc..	14	1	seq
....gauugcacuagucccggcU..	5	1	seq
....gauugcacuUgucccggcc..	12	1	seq
....gaAugcacuagucccggcc..	3	1	seq
....gauugcacGagucccggcc..	1	1	seq
....gauugcacuagAcccggcc..	1	1	seq
....Aauugcacuagucccggcc..	16	1	seq
....gauugcacuagucccggcc..	2187	0	seq
....gauugcaGuagucccggcc..	1	1	seq
....gauugcacuagucccggGc..	1	1	seq

```
                    Mature                            Star
gcguguugcucggugccagaaggucgagauuggcgcaaugcugucguccauccagauugcacuagucccggccuucuggcucugcugcucgucuucaucucgccucggcgacu
......................................................gauugcacAagucccggcc........................................ 1 1 seq
......................................................gauAgcacuagucccggccu....................................... 8 1 seq
......................................................gauGgcacuagucccggccu....................................... 1 1 seq
......................................................gauuAcacuagucccggccu....................................... 4 1 seq
......................................................gauugGacuagucccggccu....................................... 1 1 seq
......................................................gauugcacuagucccggAcu....................................... 4 1 seq
......................................................gauugcacuagucccggccu....................................... 20520 0 seq
......................................................gauugcacuagGcccggccu....................................... 1 1 seq
......................................................gauugcacuaguccUggccu....................................... 17 1 seq
......................................................gGuugcacuagucccggccu....................................... 99 1 seq
......................................................gauuUcacuagucccggccu....................................... 3 1 seq
......................................................Uauugcacuagucccggccu....................................... 25 1 seq
......................................................gauugcacuagucccgAccu....................................... 3 1 seq
......................................................gauugcacuagucAcggccu....................................... 12 1 seq
......................................................Nauugcacuagucccggccu....................................... 4 1 seq
......................................................gauugUacuagucccggccu....................................... 2 1 seq
......................................................gauugcUcuagucccggccu....................................... 2 1 seq
......................................................Cauugcacuagucccggccu....................................... 9 1 seq
......................................................gauugcacuagucccggcGu....................................... 1 1 seq
......................................................gaAugcacuagucccggccu....................................... 16 1 seq
......................................................gauugcacuagucccggccG....................................... 62 1 seq
......................................................gauugcacuaguAccggccu....................................... 9 1 seq
......................................................gauugcacuagucccggUcu....................................... 3 1 seq
......................................................gauugcacuaguccAggccu....................................... 5 1 seq
......................................................gauuCcacuagucccggccu....................................... 1 1 seq
......................................................gauugNacuagucccggccu....................................... 1 1 seq
......................................................gauugcacuagucccUgccu....................................... 1 1 seq
......................................................gauugcacuaguUccggccu....................................... 3 1 seq
......................................................gauugcacGagucccggccu....................................... 2 1 seq
......................................................gauugcacuagucUcggccu....................................... 50 1 seq
......................................................Aauugcacuagucccggccu....................................... 88 1 seq
......................................................gauugcacuagucccggcUu....................................... 4 1 seq
......................................................gauugAacuagucccggccu....................................... 11 1 seq
......................................................gauugcaAuagucccggccu....................................... 3 1 seq
......................................................gauugcacuagucccAgccu....................................... 21 1 seq
......................................................gauugcacuGgucccggccu....................................... 3 1 seq
......................................................gauugcacuagCcccggccu....................................... 1 1 seq
......................................................gauugcacuagucccggcAu....................................... 1 1 seq
......................................................gauugcacuagucGcggccu....................................... 2 1 seq
......................................................gaCugcacuagucccggccu....................................... 4 1 seq
......................................................gauugcacuagAcccggccu....................................... 11 1 seq
......................................................gauugcacAagucccggccu....................................... 6 1 seq
......................................................gauugcacuaAucccggccu....................................... 2 1 seq
......................................................gauCgcacuagucccggccu....................................... 2 1 seq
......................................................gauugcacCagucccggccu....................................... 1 1 seq
......................................................gauugcacuaguccGggccu....................................... 3 1 seq
......................................................gauugcacuagucccggccA....................................... 29 1 seq
......................................................gauugcGcuagucccggccu....................................... 16 1 seq
......................................................gauugcacUgucccggccu........................................ 50 1 seq
......................................................gUuugcacuagucccggccu....................................... 4 1 seq
......................................................gauugcacuaUucccggccu....................................... 2 1 seq
......................................................gauugcacuagucccggccC....................................... 3 1 seq
......................................................gauugcacuagucccggcGuu...................................... 2 1 seq
......................................................gauugcacuagCcccggccuu...................................... 3 1 seq
......................................................gauugcaUuagucccggccuu...................................... 3 1 seq
......................................................gauugcacCagucccggccuu...................................... 2 1 seq
......................................................gauugcacAagucccggccuu...................................... 6 1 seq
......................................................gauugcacuagucccgCccuu...................................... 3 1 seq
......................................................Aauugcacuagucccggccuu...................................... 28 1 seq
......................................................gauCcacuagucccggccuu....................................... 1 1 seq
......................................................Uauugcacuagucccggccuu...................................... 24 1 seq
......................................................gauugcacuagucccCgccuu...................................... 2 1 seq
......................................................gauugcacuagucccggUcuu...................................... 2 1 seq
......................................................gauugcacuagucccggccAu...................................... 6 1 seq
......................................................gauugcacuaCucccggccuu...................................... 1 1 seq
......................................................gauugcacuagucccUgccuu...................................... 3 1 seq
......................................................gauugcacuagucccggAcuu...................................... 4 1 seq
......................................................Nauugcacuagucccggccuu...................................... 2 1 seq
......................................................gauugcacuUgucccggccuu...................................... 2 1 seq
......................................................gauugcacuagucccgUccuu...................................... 1 1 seq
```

Mature Star			
gcguguugcucggugccagaaggucgagauuggcgcaaugcugucguccauccagauugcacuagucccggccuucuggcucugcugcucgucuucaucucgccucggcgacu			
....gauugcacuaguAccggccuu......................................	5	1	seq
....gauuAcacuagucccggccuu......................................	2	1	seq
....gauugcacuaguccAggccuu......................................	7	1	seq
....gauugcCcuagucccggccuu......................................	2	1	seq
....gauugcacuagucccggccCu......................................	5	1	seq
....gauugcacuagucccggccuA......................................	32	1	seq
....gUuugcacuagucccggccuu......................................	1	1	seq
....gauugcacuagucccggccuC......................................	2	1	seq
....gauugcacuagucccggccuu......................................	18452	0	seq
....gauugcacuagucUcggccuu......................................	14	1	seq
....gauugcacuaguccGggccuu......................................	1	1	seq
....gauugcacuagucccgAccuu......................................	2	1	seq
....gauugcUcuagucccggccuu......................................	2	1	seq
....gauugcacuagucccggccGu......................................	6	1	seq
....gauugcacuagAcccggccuu......................................	2	1	seq
....gauugcacuagucGcggccuu......................................	2	1	seq
....gauugcGcuagucccggccuu......................................	24	1	seq
....gauugcacuGgucccggccuu......................................	1	1	seq
....gauugcacuaguccUggccuu......................................	8	1	seq
....gauugAacuagucccggccuu......................................	7	1	seq
....gauugcacuagucccAgccuu......................................	8	1	seq
....gauugUacuagucccggccuu......................................	2	1	seq
....gauugcacuaAucccggccuu......................................	2	1	seq
....gaAugcacuagucccggccuu......................................	21	1	seq
....gauugcacuaguUccggccuu......................................	1	1	seq
....gauugcacuagucccggcUuu......................................	9	1	seq
....gauAgcacuagucccggccuu......................................	5	1	seq
....gauCgcacuagucccggccuu......................................	3	1	seq
....gauugcacuagucccggGcuu......................................	3	1	seq
....Cauugcacuagucccggccuu......................................	4	1	seq
....gauugcacuagucAcggccuu......................................	6	1	seq
....gGuugcacuagucccggccuu......................................	91	1	seq
....gauugcaAuagucccggccuu......................................	1	1	seq
....gauugcacuagucccggccuG......................................	17	1	seq
....gauugcacGagucccggccuu......................................	2	1	seq
....gaCugcacuagucccggccuu......................................	1	1	seq
....gUuugcacuagucccggccuuc.....................................	3	1	seq
....gauugcacuagucccggccGuc.....................................	4	1	seq
....gauugcacuaguGccggccuuc.....................................	4	1	seq
....Nauugcacuagucccggccuuc.....................................	8	1	seq
....gauugcaGuagucccggccuuc.....................................	1	1	seq
....gGuugcacuagucccggccuuc.....................................	172	1	seq
....gauugcacuagucccgAccuuc.....................................	5	1	seq
....gauugcacuagucccAgccuuc.....................................	20	1	seq
....gauugcaAuagucccggccuuc.....................................	4	1	seq
....gauugcacuagucccggccuGc.....................................	12	1	seq
....gauugcacAagucccggccuuc.....................................	9	1	seq
....gauugcacuagucccCgccuuc.....................................	3	1	seq
....gauugcacuagucccggAcuuc.....................................	5	1	seq
....gauugcacuagucccgUccuuc.....................................	2	1	seq
....gauugcacuagucccUgccuuc.....................................	2	1	seq
....gauugcacuaguccAggccuuc.....................................	12	1	seq
....gauugcacuUgucccggccuuc.....................................	1	1	seq
....gauugcacuagucccggcGuuc.....................................	1	1	seq
....gauugcacuaCucccggccuuc.....................................	1	1	seq
....Cauugcacuagucccggccuuc.....................................	6	1	seq
....gauugcacuaguccUggccuuc.....................................	27	1	seq
....gauugcacuagucccggcAuuc.....................................	6	1	seq
....gauugcaUuagucccggccuuc.....................................	7	1	seq
....gNuugcacuagucccggccuuc.....................................	1	1	seq
....gauugcacuagucccggccAuc.....................................	17	1	seq
....gauGgcacuagucccggccuuc.....................................	1	1	seq
....gauugcacuagucccggccCuc.....................................	4	1	seq
....gauugcGcuagucccggccuuc.....................................	10	1	seq
....gauugGacuagucccggccuuc.....................................	1	1	seq
....gauugcacuagucccggcUuuc.....................................	5	1	seq
....gauugcacuagucccggccuuU.....................................	1078	1	seq
....gauugcacuagucccggccuuG.....................................	2	1	seq
....gauugUacuagucccggccuuc.....................................	7	1	seq
....gauugcacuagucGcggccuuc.....................................	3	1	seq

```
                    Mature                            Star
gcguguugcucggugccagaaggucgagauuggcgcaaugcugucguccauccagauugcacuagucccggccuucuggcucugcugcucgucuucaucucgccucggcgacu
......................................................gauuCcacuagucccggccuuc.....................................    2       1      seq
......................................................gauugcacuagucccggUcuuc.....................................    6       1      seq
......................................................Aauugcacuagucccggccuuc.....................................    130     1      seq
......................................................gauugcacGagucccggccuuc.....................................    3       1      seq
......................................................gauugcacuagCcccggccuuc.....................................    4       1      seq
......................................................gauugcCcuagucccggccuuc.....................................    2       1      seq
......................................................gauuAcacuagucccggccuuc.....................................    4       1      seq
......................................................gauugAacuagucccggccuuc.....................................    23      1      seq
......................................................gauugcacuagucccggccuCc.....................................    2       1      seq
......................................................gauugcacuaguAccggccuuc.....................................    17      1      seq
......................................................gauugcacuagucccggccuuc.....................................    38696   0      seq
......................................................gauugcacuaguUccggccuuc.....................................    8       1      seq
......................................................gauugcacuagAcccggccuuc.....................................    26      1      seq
......................................................gauugcacCagucccggccuuc.....................................    5       1      seq
......................................................gauuUcacuagucccggccuuc.....................................    5       1      seq
......................................................gauugcacuagucAcggccuuc.....................................    16      1      seq
......................................................gaGugcacuagucccggccuuc.....................................    1       1      seq
......................................................gaAugcacuagucccggccuuc.....................................    34      1      seq
......................................................gaCugcacuagucccggccuuc.....................................    7       1      seq
......................................................gauugcacuaUucccggccuuc.....................................    1       1      seq
......................................................Uauugcacuagucccggccuuc.....................................    55      1      seq
......................................................gauugcUcuagucccggccuuc.....................................    8       1      seq
......................................................gauugcacuagucUcggccuuc.....................................    142     1      seq
......................................................gauugcacuagucccggccuAc.....................................    11      1      seq
......................................................gauugcacuGgucccggccuuc.....................................    8       1      seq
......................................................gauCgcacuagucccggccuuc.....................................    2       1      seq
......................................................gauugcacuagucccggGcuuc.....................................    1       1      seq
......................................................gauugcacuagucccgCccuuc.....................................    4       1      seq
......................................................gauugcacuagucccggccuuA.....................................    51      1      seq
......................................................gauugcacuagGcccggccuuc.....................................    3       1      seq
......................................................gauAgcacuagucccggccuuc.....................................    13      1      seq
......................................................gauugcacuagucccggccuucA....................................    145     1      seq
......................................................gauugcacuaAucccggccuucu....................................    1       1      seq
......................................................gauugcacuaguccUggccuucu....................................    1       1      seq
......................................................gauugcacuagucGcggccuucu....................................    1       1      seq
......................................................gauAgcacuagucccggccuucu....................................    1       1      seq
......................................................Aauugcacuagucccggccuucu....................................    3       1      seq
......................................................gauugcGcuagucccggccuucu....................................    1       1      seq
......................................................gauugcacuagucccggccuuGu....................................    26      1      seq
......................................................gauugcacuagucccggccuucG....................................    3       1      seq
......................................................gauugcacuagucccggccAucu....................................    1       1      seq
......................................................Uauugcacuagucccggccuucu....................................    2       1      seq
......................................................gauugcacuagucccggGcuucu....................................    1       1      seq
......................................................gauugcacuagucccggccuucu....................................    1398    0      seq
......................................................gauugcacuagucccggccuuUu....................................    10      1      seq
......................................................gauugcacuagucccggccuuAu....................................    2       1      seq
......................................................gauugcacuagucccgUccuucu....................................    1       1      seq
......................................................gauugcacuagucccUgccuucu....................................    1       1      seq
......................................................gauugcacuagucccggccuucC....................................    4       1      seq
......................................................gauuUcacuagucccggccuucu....................................    1       1      seq
......................................................gGuugcacuagucccggccuucu....................................    6       1      seq
......................................................gauugcacuagucccggccuCcu....................................    1       1      seq
......................................................gauugcacuagucccggccuucuU...................................    29      1      seq
......................................................gauugcacuagucccggccuucuA...................................    1       1      seq
.......................................................Guugcacuagucccggcc........................................    1       1      seq
.......................................................auugcacuagucccggccuuc.....................................    1       0      seq
.......................................................auugcacuagucccggccuucG....................................    1       1      seq
.......................................................auugcacuagucccggccuucu....................................    1       0      seq
........................................................uugcacuagucccggccu.......................................    1       0      seq
........................................................uugcacuagucccggccuuc.....................................    2       0      seq
........................................................uugcacuagucccggccuGc.....................................    2       1      seq
.........................................................ugcacuagucccggccuuU.....................................    1       1      seq
............................................................acuagucccggccuucuggcucu..............................    4       0      seq
............................................................................uggcucugcugcucgucu...................    1       0      seq
............................................................................uggcucugcugcucgucuu..................    1       0      seq
............................................................................uggcucugcugcucgucuucau...............    1       0      seq
............................................................................uggcucugcugcucgucuucauc..............    13      0      seq
............................................................................uggcucugcugcucgucuucaucucgcc.........    1       0      seq
```
